# Red blood cell deformability is diminished in patients with Chronic Fatigue Syndrome

**DOI:** 10.3233/CH-180469

**Published:** 2019-02-23

**Authors:** Amit K. Saha, Brendan R. Schmidt, Julie Wilhelmy, Vy Nguyen, Abed Abugherir, Justin K. Do, Mohsen Nemat-Gorgani, Ronald W. Davis, Anand K. Ramasubramanian

**Affiliations:** a Department of Chemical and Materials Engineering, San José State University, San José, CA, USA; b Stanford Genome Technology Center, Stanford University School of Medicine, Palo Alto, CA, USA

**Keywords:** Chronic Fatigue Syndrome, microfluidics, cell deformability, red blood cells

## Abstract

**BACKGROUND:**

Myalgic encephalomyelitis/Chronic Fatigue Syndrome (ME/CFS) is a poorly understood disease. Amongst others symptoms, the disease is associated with profound fatigue, cognitive dysfunction, sleep abnormalities, and other symptoms that are made worse by physical or mental exertion. While the etiology of the disease is still debated, evidence suggests oxidative damage to immune and hematological systems as one of the pathophysiological mechanisms of the disease. Since red blood cells (RBCs) are well-known scavengers of oxidative stress, and are critical in microvascular perfusion and tissue oxygenation, we hypothesized that RBC deformability is adversely affected in ME/CFS.

**METHODS:**

We used a custom microfluidic platform and high-speed microscopy to assess the difference in deformability of RBCs obtained from ME/CFS patients and age-matched healthy controls.

**RESULTS AND CONCLUSION:**

We observed from various measures of deformability that the RBCs isolated from ME/CFS patients were significantly stiffer than those from healthy controls. Our observations suggest that RBC transport through microcapillaries may explain, at least in part, the ME/CFS phenotype, and promises to be a novel first-pass diagnostic test.

## 1 Introduction

Myalgic encephalomyelitis/Chronic Fatigue Syndrome (ME/CFS) is a multi-systemic, debilitating illness of unknown etiology affecting millions of individuals worldwide [1], with capacity to persist for decades. Although an abnormal profile of circulating proinflammatory cytokines, and the presence of chronic oxidative and nitrosative stresses have been identified and correlated with severity in ME/CFS [2, 3], there are no molecular or cellular biomarkers of the disease. Consequently, without any definitive or even non-specific diagnosis, the disease may be misdiagnosed or remain in disguise for a long time. Of interest, the shape and size of the red blood cells (RBC) change appreciably in response to oxidative and inflammatory stresses [4, 5]. The usual shape of RBC is biconcave discoid, which provides a specific surface area-to-volume ratio that facilitates large reversible deformations to any arbitrary shape, enabling travel through microvessels for optimal tissue oxygenation [6–8]. The deformation of RBCs to physical forces has been investigated as a possible diagnostic biomarker for sickle cell disease [9], malaria [10], and Gulf War Illness [11]. In this study, we tested the hypothesis that deformability of RBCs is significantly altered in ME/CFS compared to normal population, using a high-throughput microfluidic device and high-speed microscopy [10].

## 2 Materials and methods

**Fig.1:**
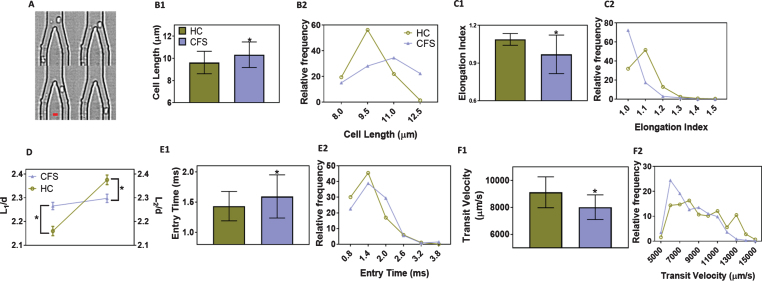
Red blood cell (RBC) motion in microfluidic channels. (A) Representative traces of a RBC before, during and after entry through the test channels (5μm×5μm). Scale bar is 5μm.; (B1) Cell length (major axis) and (B2) Distribution of cell lengths, of RBCs before entering the test channels; (C1) Elongation index and (C2) Distribution of elongation indices of deformed RBCs inside the test channels; (D) Comparison of scaled average lengths of undeformed (L1) and deformed (L2) RBCs from healthy controls and ME/CFS patients. The lengths were scaled to channel width (d = 5μm); (E1) Entry times and (E2) Distribution of entry times, i.e., time taken by RBCs to fully enter the test channels; (F1) Transit velocity and (F2) Distribution of transit velocities, based on the time taken by RBCs to travel 100μm distance inside the test channels. The results are based on at least two separate microfluidic devices per donor, and at least 100 cells were tested per device. The results are shown as mean±SD. *denotes significant differences in comparison to healthy samples, based on 2-way ANOVA with Tukey’s *post hoc* analysis (*p* < 0.0001; *n* = 16 pairs). For distribution, the results are presented as histogram with relative frequency in percentage. HC: RBCs from healthy donors; CFS: RBCs from ME/CFS patients.

ME/CFS patients previously diagnosed by a physician using the Canadian Consensus Criteria [11] were selected for the study (*n* = 16 pairs of patients with age matched healthy controls). Written consent was obtained following Stanford IRB-40146. The mean ages were 52±7.7 for the healthy controls (HC) and 52±6.7 for the ME/CFS patients. The disability scores were 94±5.4% for the HC and 44±19% for the patients. The female to male ratio was 9:7 for the healthy group and 11:5 for the ME/CFS group. All experiments were performed within 3–6 h of blood draw (all the time at room temperature), and the exact timing did not affect our results [12]. Whole blood was centrifuged at 250× G for 20 minutes to obtain the RBC fraction. A dilute suspension (∼1% hematocrit) of 10^7^ RBCs/ml in PBS (pH 7.4) was perfused through microfluidic channels using a custom vacuum-based delivery system at a pressure of -2 psi. The microfluidic channels were made of polydimethylsiloxane (PDMS) using Silicon master molds, as described previously in detail [10]. The channels were washed with 0.1% pluronic F127 (in PBS), before the cells were perfused, in order to prevent non-specific cell adhesion. RBCs moved through channels of progressively narrower cross section, starting from a width of 1 mm tapering down to 20μm before entering the 5μm× 5μm test channels. The flow of RBCs through the 5μm test channels was visualized at 40× magnification and 4000 fps. The videos were analyzed off-line to quantify three parameters as a measure of deformability: entry time (time taken by the cells to fully enter the 5μm test channels); transit velocity (velocity of RBCs traveling a distance of 100μm through the test channels); and elongation index (ratio of the length of RBCs in the test channel to the length before entry into the test channel) (Fig. 1A).

## 3 Results and discussion

We first validated the ability of the microfluidic platform to detect changes in RBC deformability. RBCs from healthy volunteers were treated with 0.01% glutaraldehyde and perfused, since this treatment has been shown to have a cell stiffening effect [13, 14]. Consistent with published studies, we observed that the cells show significantly higher entry time (∼49%), lower transit velocity (∼47%) and lower elongation index (∼7%), as compared to untreated RBCs (*p* < 0.0001, *n* = 3). Next, we perfused RBCs from healthy controls and CFS patients through the microfluidic channels. We observed that the diameter of RBCs prior to entering the test channels in ME/CFS patients was ∼10% more compared to HC. The RBCs from ME/CFS patients showed a broader distribution compared to the HC, and a notable population of the RBCs (∼20%) from ME/CFS patients were unusually bigger (>12μm), suggesting the varying impact of the disease on RBCs isolated from different patients (Fig. 1B). Next, we observed ∼15% decrease in the elongation index of RBCs from ME/CFS patients compared those from HC. The distribution of elongation indices shows that most of the cells (>70%) from ME/CFS patients do not deform after entering the test channels, while majority of cells from HC deformed at least 10–25% upon entering the test channels (Fig. 1C). By comparing the change in cell diameters normalized to the width of the test channel before (*L*_1_/*d*) and after (*L*_2_/*d*) entry, Fig. 1D clearly demonstrates that RBCs from ME/CFS patients deform 7-fold less compared to those from the HC. The cells from ME/CFS patients take ∼14% more time to enter the test channels (Fig. 1E), and upon entering the channels, travel ∼18% slower than those from HC (Fig. 1F). The distributions for entry time and transit velocity are skewed towards lower values for HC and towards higher values for those for ME/CFS patients. Because of the nature of the disease, often most patients were prescribed a wide range of medications and supplements depending on their particular symptoms and condition. For instance, these medications and supplements include Rituxan and loratadine or low-dose naltrexone, Vitamin D3 etc. Based on extensive literature search, we found only one of the drugs, colchicine, which was prescribed to one patient, has been shown to slightly affect cellular deformability [15, 16]. However, the deformability parameters for this particular patient were well within the range for other patients. The patients and volunteers identified themselves as non-smokers [17].

Together, the various estimates show that the RBCs in ME/CFS patients are significantly less deformable than those of HC. Our observations may provide an opportunity for a first-pass diagnostic test for ME/CFS with a finger-prick blood sample, with a caveat that the test may be non-specific as other inflammatory conditions or existing co-morbidities may alter RBC deformability, and may have to be placed in context with other clinical presentations. We speculate that the larger and less deformable RBCs in ME/CFS patients may partly explain the musculoskeletal pain and fatigue in the pathophysiology of ME/CFS due to impaired microvascular perfusion and tissue oxygenation. Of note, it was previously reported that no significant difference in average deformability was observed between erythrocytes obtained from healthy controls and ME/CFS patients, as measured by ektacytometer [18]. This apparent contradiction can be because of the lower sensitivity of the ektacytometer, which reports the deformability based on time and population averages indirectly from light scattering, while as seen from this work, the deformability is best measured directly on an individual cell basis.

## Authorship contributions

A.K.S., M.N.G., R.W.D., and A.K.R designed the study, analyzed the data and wrote the manuscript. J.W. arranged and collected blood samples from ME/CFS patients, and the matching controls. A.K.S., B.R.S., A.A., V.N., and J.K.D performed microfluidics experiments.

## Disclosure of conflicts of interest

The authors declare no conflict of interest.
